# Extracellular vesicle biopotentiated hydrogels for diabetic wound healing: The art of living nanomaterials combined with soft scaffolds

**DOI:** 10.1016/j.mtbio.2023.100810

**Published:** 2023-09-22

**Authors:** Zhenzhen Yan, Tinglin Zhang, Yuxiang Wang, Shichu Xiao, Jie Gao

**Affiliations:** a Department of Burn Surgery, The First Affiliated Hospital of Naval Medical University, Shanghai, 200433, People's Republic of China; b Changhai Clinical Research Unit, The First Affiliated Hospital of Naval Medical University, Shanghai, 200433, People's Republic of China

**Keywords:** Diabetic wounds, Extracellular vesicle, Hydrogel, Design strategy, Mechanism

## Abstract

Diabetic wounds (DWs) pose a major challenge for the public health system owing to their high incidence, complex pathogenesis, and long recovery time; thus, there is an urgent need to develop innovative therapies to accelerate the healing process of diabetic wounds. As natural nanovesicles, extracellular vesicles (EVs) are rich in sources with low immunogenicity and abundant nutritive molecules and exert potent therapeutic effects on diabetic wound healing. To avoid the rapid removal of EVs, a suitable delivery system is required for their controlled release. Owing to the advantages of high porosity, good biocompatibility, and adjustable physical and chemical properties of hydrogels, EV biopotentiated hydrogels can aid in achieving precise and favorable therapy against diabetic wounds. This review highlights the different design strategies, therapeutic effects, and mechanisms of EV biopotentiated hydrogels. We also discussed the future challenges and opportunities of using EV biopotentiated hydrogels for diabetic wound healing.

## Graphical abstract

Design strategies and mechanisms of EV biopotentiated hydrogels for promoting diabetic wound healing. EV, extracellular vesicle. Created by BioRender. com.

**Figure:**
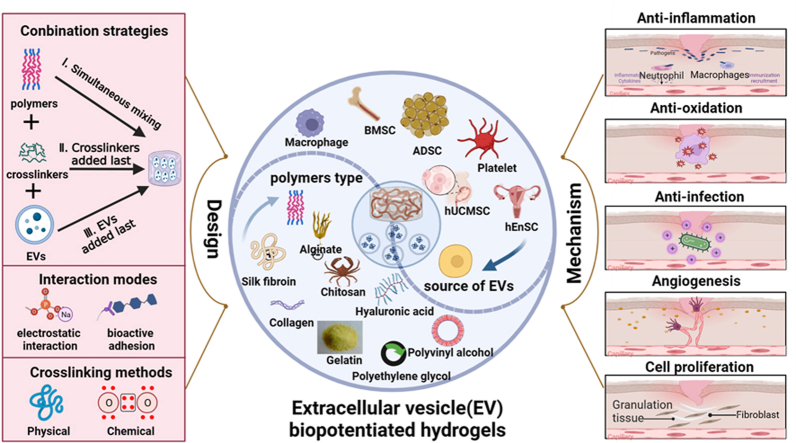

## 1 Introduction

Diabetes mellitus, a metabolic disease characterized by hyperglycemia resulting from insufficient insulin secretion and/or insulin resistance, is highly prevalent worldwide. Currently, nearly 500 million people worldwide have diabetes, and this number is projected to increase to 642 million by 2040 [1,2]. Diabetic wounds (DWs) are one of the most common complications in diabetes patients, and 2.5%–15% of healthcare budgets worldwide are spent on diabetes and diabetic wound management annually [[3], [4], [5]]. The complex pathogenesis of diabetic wounds involves persistent inflammatory responses, microbial infections, high oxidative stress, vascular abnormalities, and impaired epithelialization [[6], [7], [8]]. The traditional management for DWs primarily includes wound debridement, offloading, antibiotic therapy, and topical dressings. Among these therapeutic strategies, dressing closure plays a pivotal role in the treatment of DWs. Nonetheless, conventional dry dressings like gauze exhibit limitations, including wound dehydration, hindered epithelial cell migration, and insufficient protection against bacterial intrusion. Furthermore, these dressings often adhere to developing granulation tissue, causing secondary wound damage during dressing changes. To address these challenges, innovative wet healing dressings have emerged, encompassing hydrogels, films, hydrocolloids, sponges, etc. [9,10].

Hydrogels are hydrophilic three-dimensional structures formed by the physical or chemical crosslinking of polymer chains. With excellent biocompatibility and water retention properties, hydrogels have been regarded as ideal functional wound dressings that conform to the concept of moist wound healing [11,12]. Hydrogels are promising for a wide range of applications, including tissue regeneration, wound healing, and drug delivery. As wound dressings, hydrogels possess inherent antibacterial properties and good adhesive strength, serving as biological barriers that isolate bacteria, maintain a moist environment, absorb wound exudates, and promote autolytic debridement [13,14]. Furthermore, injectable hydrogels formed *in situ* possess self-healing abilities, enabling the restoration of their structural stability and original properties after external damage. They are suitable for deep, irregularly shaped skin defects, achieving the desirable effect of conforming to the wound surface while avoiding adhesion, making them an ideal wound dressing [15]. However, the lack of active molecules in hydrogels restricts their application, as they cannot dynamically provide the cells and growth factors required for different stages of wound healing. In recent years, significant advancements have been made in augmenting the biological functionalities and mechanical characteristics of hydrogels through the integration of bioactive elements, including growth factors, genetic materials, stem cells, and extracellular vesicles (EVs). This innovative strategy offers the potential for achieving precise and sustained modulation of the microenvironment within diabetic wound sites [16].

Extracellular vesicles are heterogeneous nano-sized vesicles composed of a lipid bilayer membrane. They are laden with diverse biological molecules, such as lipids, proteins, nucleic acids (DNA, mRNA, and ncRNA), and metabolites. EVs are involved in intercellular communication by transferring their cargo between the donor and target cells, which is mediated by their ability to cross biological barriers [17]. Recently, EVs have garnered extensive attention as cell-free therapies in regenerative medicine. Based on their origin, EVs can be categorized into three main subtypes: exosomes (40–160 nm), microvesicles (100–1000 nm), and apoptotic bodies (50–5000 nm). EVs are involved in a myriad of pathological and physiological processes in diseases owing to their abundant sources, natural transport vehicles, and immune tolerance. In the context of wound healing and tissue regeneration, EVs play pivotal roles by promoting essential processes such as cell proliferation, autophagy, collagen maturation, epithelial-mesenchymal transition, and angiogenesis [18,19]. However, direct administration of EVs to wounds poses several challenges, such as a low survival rate and clearance by the reticuloendothelial system. Systemic injection of EVs results in rapid clearance from circulation, whereas local injection results in internalization by surrounding tissues, thereby restricting the therapeutic impact of EVs [20,21]. To overcome these limitations and achieve stable and durable therapeutic effects, it is necessary to use long-acting and sustained-release biomaterials to encapsulate EVs.

Hydrogels are regarded as excellent candidates for loading EVs because they can provide an extracellular matrix (ECM)-like microenvironment, mechanical support, and immune protection for EVs [22]. Composite hydrogels loaded with EVs have an enormous potential to improve wound healing [23]. Hydrogels not only provide a protective scaffold for EVs but also prolong their half-life and bioactivity, realizing the controlled release of bioactive molecules [24]. Furthermore, EVs exhibit favorable reparative effects through the transmission of active molecules at different stages of skin wound healing [25]. Additionally, EVs can accomplish the targeted delivery of molecules or drugs through homing effects [26]. The combination of the hydrogel and an EV compensates for their respective shortcomings and demonstrates a synergistic action. Therefore, EV delivery using hydrogel scaffolds is a promising cell-free therapy for wound healing and tissue regeneration.

In the emerging field of composite hydrogels loaded with EVs, despite some reviews discussing their progress in tissue regeneration, including promotion of wound healing, regulation of osteogenic differentiation, spinal cord injury repair, cardiovascular function restoration, and immune modulation. However, to the best of our knowledge, a comprehensive systematic review of composite hydrogels loaded with EVs applied to diabetic wound repair is currently lacking [23,27]. Therefore, this review summarizes the design strategies for enhancing the efficiency of EV binding to hydrogels based on different crosslinking methods and elaborates on the therapeutic effects and molecular mechanisms of EV-loaded composite hydrogels in diabetic wound healing. Finally, we discussed the prospects and challenges associated with the clinical translation of EV-loaded composite hydrogels for diabetic wound repair.

## 2 Design strategies for composite hydrogels loaded with extracellular vesicles

EVs have great potential for the diagnosis and treatment of various diseases because of their wide availability, low immune rejection, and the ability to carry multiple bioactive molecules [28]. However, most intravenously administered EVs have a half-life of less than 30 min in circulation, and locally administered EVs are cleared from the wound bed as early as the second day [29]. Tissue engineering research has revealed that loading EVs into hydrogels not only increases their retention at the target tissue site but also achieves sustained and controlled release of the active ingredients by adjusting the degradation rate of hydrogels, thereby exerting more stable and persistent therapeutic effects [30]. Moreover, hydrogels can act as biological scaffolds, providing the matrix necessary for tissue repair and a favorable microenvironment for wound healing [31].

### 2.1 Combination strategies for extracellular vesicles and hydrogels

Currently, there are three methods for loading EVs into hydrogels, which differ in the order in which EVs are added to the hydrogel matrix. The first is the simultaneous preparation of hydrogels by mixing purified EVs, polymers, and crosslinkers suitable for filling deep cavities or irregularly shaped wounds [32]. This method involves injecting the mixture directly into the target site via *in situ* gelation induced by ultraviolet (UV) radiation, ion exchange, pH changes, temperature changes, and other mechanisms [33]. The second step is the binding of the EVs and polymers, followed by the addition of a crosslinking agent to induce gelation, which is based on the covalent crosslinking of the active precursor. Hydrogels obtained using this method have tunable mechanical properties and degradation rates. However, when crosslinking agents are added, they can be cytotoxic to biomolecules [34]. The third step is to premix the polymers and crosslinkers to form a stable dry hydrogel structure, which is then immersed in an aqueous solution of EVs for full absorption, and dried to obtain a composite hydrogel loaded with EVs [35]. This method is more advantageous because the performance of EVs is not affected by the polymerization conditions but requires that the pore size of the hydrogel be larger than the EVs to be encapsulated.

To improve the loading and binding efficiency of EVs in hydrogels, two types of interactions, electrostatic interactions and bioactive adhesion between EVs and hydrogels, can be exploited. First, the negatively charged properties of EVs (negatively charged phospholipid membrane and sugar coat residues) can attract positively charged cationic polymer hydrogels, greatly increasing the loading efficiency of EVs [36]. For example, stable hydrogel structures loaded with exosomes have been obtained by exploiting the electrostatic binding interaction between the positive NH2 charge of chitosan and exosomes [37]. In addition, Diomede et al. used polyethyleneimine (PEI), a positively charged polymer, to wrap gingival mesenchymal stem cell-derived exosomes (GMSC-Exos), which significantly increased the adhesion and retention time of three-dimensional (3D) engineered scaffolds and collagen membranes [38]. Second, the bioactive adhesion effect between the adhesion molecules expressed on EVs and hydrogels composed of natural ECM-based components can also be utilized to increase the loading efficiency of EVs in hydrogels. For example, EVs can bind to fibronectin and collagen through integrins or to hyaluronic acid (HA) through CD44 [39,40]. Additionally, the EV membrane surface can be anchored by peptide modification for binding to the hydrogels. EV membrane surfaces can bind to hydrogels through anchoring peptide modifications. The peptide sequence CP05 (CRHSQMTVTSRL) specifically recognizes and captures the exosome marker CD63. Ma et al. effectively introduced exosomes into porcine small intestinal submucosal hydrogels by constructing a multifunctional fusion peptide composed mainly of collagen and rich in several bioactive factors. The fusion peptide consisted of collagen-binding domains (TKKTLRT and DARKSEVQK) and the exosome-capturing peptide CP05. The results showed that the fusion peptide enhanced the retention and stability of exosomes in hydrogels [41].

The potency of EVs relies heavily on the design and functionality of the hydrogels. Factors such as pore size, crosslinking density, mechanical strength, biodegradability, crosslinking method, and material source of hydrogels may affect their performance, thereby affecting the release and activity of EVs. Recent studies have revealed an intriguing paradox regarding the diffusion behavior of exosomes regulated by hydrogel stiffness—compared to soft hydrogels, hard hydrogels release exosomes faster *in vitro* but slower *in vivo* [42]. The pore size of the hydrogel is crucial because the weak physical binding of exosomes makes it difficult for them to enter small pores, whereas large pores make them susceptible to leakage. In addition, a higher hydrogel crosslinking density leads to a smaller mesh size, allowing for a longer retention time and slower diffusion of EVs. The biodegradation rate is a critical factor in the application of hydrogels as tissue engineering scaffolds for skin injury treatment. Therefore, the release and functional characteristics of embedded EVs can be controlled by adjusting the swelling and degradation rates of hydrogel matrices [43]. The crosslinking method is also an important factor affecting the gelation rate of hydrogels. Born et al. incorporated EVs from mesenchymal stem cells into gelatin methacrylate (GelMA) for 3D printing and reduced the initial burst release of EVs by increasing the concentration of the crosslinking agent during material crosslinking, thereby achieving sustainable release of EVs [44]. Hydrogels with highly tunable properties based on different crosslinking methods can meet the requirements of different skin injuries and can be classified as physically and chemically crosslinked hydrogels for EV delivery.

### 2.2 Effects of crosslinking methods on hydrogel-EV composites

#### 2.2.1 Physical crosslinking

Physical crosslinking refers to the formation of polymers through intermolecular, non-covalent interactions between monomers. Noncovalent hydrogels are primarily based on electrostatic bonding [45], hydrogen bonding [46], and hydrophobic interactions [47]. This method is easy to perform and does not require crosslinking agents. The synthesized hydrogels have good degradability, shear-thinning behavior, and excellent environmental responsiveness, making them a highly promising area for hydrogel development. However, hydrogels prepared via physical crosslinking typically exhibit poor mechanical properties, low long-term stability, and susceptibility to degradation.

Electrostatic binding can be defined as the interaction between a polyelectrolyte and a substance with an opposite charge. For example, hydrogels formed by electrostatic attraction between positively charged chitosan and negatively charged HA can act as carriers for keratinocyte growth and promote wound healing [48]. In addition, Nooshabadi et al. prepared a chitosan-glycerol hydrogel loaded with human endometrial stem cell-derived exosomes via electrostatic interactions between the positive amino charge of chitosan and the negative charge of the phosphate group of glycerol. Chitosan-glycerol-Exo composite hydrogels significantly promoted epidermal regeneration, granulation tissue, and new capillary formation with excellent wound-healing ability [37].

Hydrogen bonding is a specific dipole–dipole interaction between hydrogen and a negatively charged atom (e.g., oxygen, nitrogen, or fluorine). Hydrogen bonding has been widely used in the crosslinking of hydrogels because of its unique directionality, tunability, and specificity [49]. For instance, polyvinyl alcohol (PVA) molecular chains contain many hydrophilic groups that can form hydrogen bonds with alginate and crosslink to form hydrogels with good permeability in the presence of calcium chloride. Notably, this hydrogel represents a dual-crosslinked system in which the negatively charged –COO– groups in alginate effectively bind to Ca^2+^, establishing an additional level of interaction based on electrostatic forces. Based on this mechanism, researchers designed a PVA/alginate composite hydrogel (exo@H) loaded with human umbilical cord mesenchymal stem cell-derived exosomes (hUCMSC-exos) for wound healing in diabetic rats. Notably, exo@H demonstrated a remarkable capacity to stimulate the proliferation, migration, and angiogenesis of human umbilical vein endothelial cells (HUVECs), effectively expediting the healing of diabetic wounds [50].

Hydrophobic interactions are a vital means of noncovalent binding in polymers. Amphiphilic block copolymers composed of hydrophilic and hydrophobic segments self-assemble in aqueous solution to form a reticulated hydrogel structure by associative clustering [51]. Pluronics, which are representative triblock copolymers composed of poly (ethylene oxide)-poly (propylene oxide)-poly (ethylene oxide), exhibit reversible thermoresponsive properties, transitioning from a liquid state with pronounced hydrophilicity at lower temperatures (≤10 °C) to a gel state with enhanced hydrophobicity at higher temperatures (20–37 °C) [52]. Among these, Pluronic F-127 (PF-127) has gained Food and Drug Administration (FDA) approval for human applications because of its injectability, biocompatibility, and thermosensitive properties, making it a versatile choice for drug delivery and controlled-release strategies [53]. Yang et al. constructed a PF-127-loaded hUCMSC-exos composite hydrogel for the treatment of chronic diabetic wounds. Meticulous *in vitro* and *in vivo* evaluations convincingly demonstrated that PF-127 preserved biological activity and sustained the release of exosomes, concurrently modulating the ratio of type I to type III collagen, thus fostering scarless wound healing [54].

#### 2.2.2 Chemical crosslinking

Chemical crosslinking refers to the formation of covalent crosslinks via monomer polymerization or functional group reactions [55]. The main chemical crosslinking methods include click chemistry [56], Schiff base reactions [57], Michael addition [58], photocrosslinking [59], and enzyme-catalyzed crosslinking [60]. Chemical crosslinking has the advantages of finely controlled crosslinking and a high crosslinking strength. However, it is important to consider the potential limitations associated with chemical crosslinking, such as the decreased toughness and increased biotoxicity of the obtained hydrogels.

Click chemistry is a cost-effective method for the rapid synthesis of diverse compounds with the advantages of mild reaction conditions, high selectivity, straightforward purification, and excellent yield [61]. Shen et al. devised a bilayer hydrogel system composed of thiolated sodium alginate (SA) and polyethylene glycol (PEG) diacrylate to achieve sequential release of small extracellular vesicles (sEVs). In the initial stages of wound healing, the lower layer of the hydrogel releases unmodified sEVs, promoting endothelial cell proliferation and migration. Subsequently, during the proliferative remodeling phase, miR-29b-sEVs are released from the upper layer of the hydrogel to promote scar-free wound healing by inhibiting excessive deposition of blood vessels and collagen [62].

The Schiff base reaction, which is characterized by the reversible formation of imine bonds between amino and aldehyde groups, offers a rapid and efficient approach for hydrogel synthesis. This reaction is rapid and can form hydrogels within a few minutes. The reversibility of the imine bonds imparts self-healing properties to the hydrogel, which can rapidly recover its structure and function after external damage, thereby demonstrating its stability [63]. Wang et al. synthesized a multifunctional hydrogel (FEP) via a Schiff base reaction between PEI grafted onto Pluronic F127 and the aldehyde pullulan, which was used to load adipose-derived mesenchymal stem cell-derived exosomes (ADSC-exos). The dynamic Schiff base bond in the hydrogel allowed two pieces of scaffold dressings to be placed together or new scaffolds to be added to the defective surface, which achieved complete healing within 15 s. In addition, *in vitro* experiments showed that FEP hydrogel-loaded exosomes could be released continuously for 3 weeks with changes in wound pH, exhibiting excellent ability to promote proliferation, migration, and tube formation of HUVECs [64].

Michael addition reactions involve the nucleophilic addition of negative carbon ions or nucleophilic reagents with unsaturated carbonyl compounds. This methodology offers exceptional advantages in terms of efficiency, selectivity, minimal generation of byproducts, and the absence of catalysts and coupling agents, thereby representing an appealing approach in synthetic chemistry [65]. In a recent study, Jiang et al. developed an intelligent hydrogel, denoted as ADSC-exo@MMP-PEG, crafted through the Michael addition reaction between a maleimide group (4-arm-PEG-MAL) and a thiol group (PEG-dithiol, matrix metalloproteinase [MMP] degradable peptide, and exosomes). This innovative hydrogel exhibited remarkable potential for treating diabetic wounds and showed substantial promise in the field of wound healing [66].

Under UV or visible-light irradiation, compounds containing photosensitive functional groups can be cross-linked intra- or intermolecularly to form three-dimensional networks of hydrogels. Photocrosslinking is temporally and spatially controllable, thereby presenting a convenient approach for diverse applications [67,68]. Henriques et al. mixed thiolated HA- and photocleavable linkers-pretreated EVs using a UV-initiated copolymerization reaction to prepare a photosensitive hydrogel. *In vivo* assessments of the distribution and stability of EVs were performed using the Forster resonance energy-transfer system, which revealed that EVs bound to the photo-triggered hydrogel exhibited enhanced stability and enabled sustained release at the wound site [69].

Enzymatic crosslinking is a chemoselective approach that utilizes enzymes as catalysts and is characterized by their remarkable catalytic properties, high selectivity, and mild reaction conditions [70]. Proteinaceous polymers are suitable for enzyme-catalyzed gelation reactions owing to the presence of active biomolecules, and their products are mostly biodegradable [71]. Transglutaminase (TGase) is an enzyme that naturally catalyzes the formation of heteropeptide bonds between lysine ε-amines and glutamine side chain amides and can be used to generate intermolecular crosslinks between soluble fibrinogen molecules [72]. Recently, Xu et al. assessed the potential of TGase-mediated crosslinking to fabricate a novel rhCol III hydrogel aiming to precisely regulate the release of EVs derived from hUC-MSCs. Given the presence of γ-glutamyl and ε-amine groups on the membrane surface of EVs, TGase enabled secure immobilization onto the rhCol III hydrogel matrix, thereby safely and effectively avoiding the abrupt release of EVs. Comprehensive *in vitro* and *in vivo* investigations collectively revealed this rhCol III-EV hydrogel system could effectively attenuate the inflammatory response of the wound and facilitate cellular migration and neovascularization, thus accelerating the regeneration of the traumatized tissue [73].

The selection of appropriate crosslinking methods plays a critical role in determining the properties and performance of hydrogel-EV composites (Table 1). Physical crosslinking allows the entrapment of EVs within the hydrogel matrix without compromising their structural integrity and bioactivity. Chemical crosslinking strategies can effectively immobilize EVs within a hydrogel network, thereby enhancing their stability and controlled release. Future research should focus on the development and optimization of physicochemical hybridization methods for binding EVs within hydrogel matrices. Hybrid hydrogel-EV composites can be tailored by combining physical and chemical crosslinking strategies to enhance encapsulation efficiency, tunable release kinetics, mechanical strength, and biocompatibility for diabetic wound healing applications.

**Table 1:** Characteristics of hydrogels formed based on different crosslinking methods.

Crosslinking methods		Crosslinking mechanism	Crosslinking characteristics	Ref.
Physical crosslinking	hydrogen bond	a dipole-dipole interaction between a negatively charged atom and a hydrogen atom bound to another negatively charged atom	reversible, poor mechanical strength, viscoelastic, and self-healing properties	[74]
	electrostatic interactions	polymers and oppositely charged small molecules or polymers are attracted to each other	reversible responsiveness and excellent mechanical properties	[75]
	hydrophobic interactions	spontaneous aggregation of hydrophobic groups to form hydrophobic association microregions	reversible, temperature sensitivity, and injectability	[54,76]
Chemical crosslinking	click reactions	addition reactions between sulfhydryl groups and double bonds	Irreversible, high selectivity, and mild reaction conditions	[62]
	Schiff base reaction	formation of reversible imine bonds between amino and aldehyde groups	reversible, rapid reaction, and self-healing properties	[35,64,77,78]
	Michael addition	nucleophilic addition reactions of carbon negative ions or nucleophilic reagents with unsaturated carbonyl compounds	irreversible, selectivity, fewer byproducts, and no catalyst required	[66]
	photocrosslinking	compounds containing photosensitive groups cross-linked under light irradiation	irreversible and temporally and spatially controllable	[69,79]
	enzymatic crosslinking	chemical reactions with enzymes as catalysts	irreversible, remarkable catalytic properties, and high selectivity	[73]

## 3 Role of composite hydrogels loaded with extracellular vesicles in wound healing

Hydrogel materials include natural and synthetic polymers, each of which possesses distinct biological functionalities. Natural hydrogels such as polysaccharide and protein hydrogels demonstrate exceptional capabilities in promoting cell adhesion, migration, and differentiation owing to their remarkable biocompatibility, biodegradability, and immunogenicity suppression [80]. Nonetheless, natural hydrogels encounter challenges concerning their mechanical properties and reproducibility, which can be effectively overcome through the development of synthetic hydrogels such as PEG and PVA hydrogels [81]. In the following section, we discuss several prevalent natural and synthetic hydrogels along with the wound-healing effects achieved by loading EVs into these hydrogels.

### 3.1 Polysaccharide-based hydrogels

Polysaccharides, which are abundant in the natural environment, exhibit remarkable properties such as biocompatibility, biodegradability, and non-toxicity. Polysaccharide degradation products pose no harm to the human body and can be readily metabolized. Notably, several polysaccharide-based hydrogels, including SA, HA, and chitosan, have received FDA approval for use in wound-healing therapies. Furthermore, extensive research has been conducted to explore the potential of these polysaccharide-based hydrogels as platforms for the localized delivery of EVs.

**Fig. 1:**
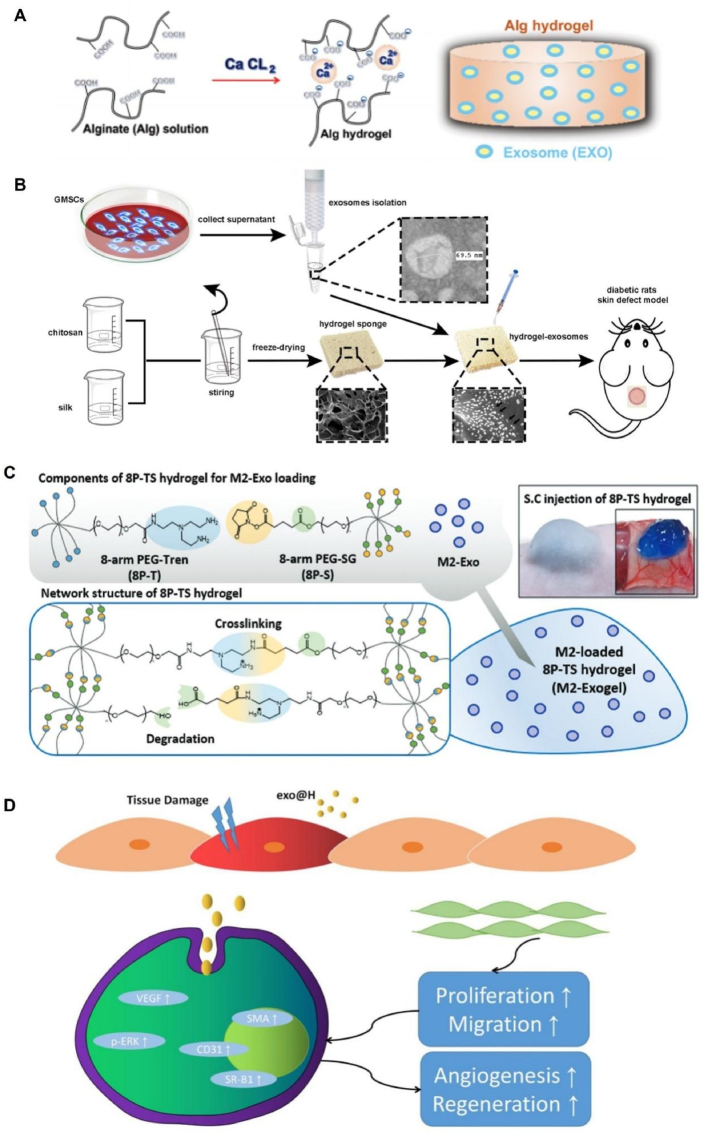
Synthesis of hydrogels from different material sources. (A) Sodium alginate hydrogel loaded with ADSC-EXOs. Reproduced with permission from Ref. [85], Copyright © 2019 Wiley Periodicals, Inc. (B) The combination of GMSC-derived exosomes and chitosan/silk protein hydrogel. Reproduced with permission from Ref. [35], Copyright © 2017 Frontiers. (C) Preparation process of PEG hydrogel loaded with M2-Exos (Exogel). Reproduced with permission from Ref. [108], Copyright © 2020 Elsevier B.V. (D) Schematic illustration of the exo@H pro-healing mechanism. Reproduced with permission from Ref. [50], Copyright © 2022 Wiley-VCH GmbH. ADSC-EXOs, adipose-derived mesenchymal stem cell-derived exosomes; GMSC, gingiva-derived mesenchymal stem cells.

Sodium alginate, derived from brown algae, is a linear polysaccharide and a derivative of alginate that is widely available and has excellent biosafety [82]. In dried alginate dressings, SA can absorb wound fluid and form a hydrogel that maintains a moist wound environment. This hydrogel property aids in minimizing bacterial infection and promotes the formation of granulation tissue within the wound [83]. The rapid gel formation ability of SA via an ion-exchange reaction with highly valent cations has led to its use as a delivery vehicle for diverse active molecules [84]. Shafei et al. mixed an ADSCs-exos solution with an SA solution and then added calcium chloride solution to form a hydrogel. (Fig. 1A). It was observed that exosomes were slowly released with the degradation of the hydrogel, and the exosome release rate reached more than 50% after 72 h and was completely released after 172 h. The results of the *in vivo* experiments showed that collagen synthesis and neovascularization were improved in the sodium alginate hydrogel group alone compared to the control group. However, the sodium alginate composites group with controlled release of exosomes exhibited better and faster wound closure in rat models [85].

Hyaluronic acid represents the simplest glycosaminoglycan and is composed of β-d-glucuronic acid and N-acetyl-β-d-glucosamine units linked via β-1,4 glycosidic bonds [86]. HA is extensively distributed in diverse tissues of living organisms and possesses the inherent advantage of being non-immunogenic, allowing easy loading of cellular or bioactive molecules. The exceptional water retention properties of HA hydrogels make them versatile materials for tissue regeneration, drug delivery, and wound management [87]. Furthermore, owing to its intrinsic rheological attributes, functionalized HA has emerged as a promising bioink for 3D printing applications. In a recent study, methacrylic acid-HA (MeHA) was used as a bioink to fabricate advanced smart dressings using cutting-edge 3D printing technology. Empirical evidence has substantiated the superior mechanical properties, controlled swelling kinetics, prolonged degradation profiles, and favorable biocompatibility of 3D-printed MeHA patches. Notably, compared with their non-exosome-loaded counterparts, MeHA patches incorporating human mesenchymal stem cell-derived exosomes exhibited pronounced enhancement in the proliferation, migration, and angiogenic potential of human fibroblasts and human endothelial cells [79].

Chitosan, a cationic polysaccharide derived from chitin, is the sole basic polysaccharide found in nature and is composed of diglucosamine and N-acetylglucosamine units. Notably, chitosan-based hydrogels have garnered significant attention because of their inherent biocompatibility, nontoxicity, antimicrobial properties, and hemostatic potential, rendering them highly desirable for multifaceted applications. Specifically, these hydrogels serve as exceptional dressings for traumatic wounds and offer a versatile platform for the efficient delivery of diverse substances [88,89]. Abolgheit et al. evaluated the efficacy of chitosan-based hydrogels loaded with bone marrow-derived mesenchymal stem cells (BM-MSCs) versus those loaded with BM-MSC-secreted EVs in promoting wound healing. Experimental results showed that collagen scaffolds introduced with chitosan acquired better mechanical properties and inherent pro-wound healing effects. Notably, scaffolds incorporating EVs exhibited enhanced collagen deposition and displayed a more regular alignment pattern than those loaded with MSCs, thereby underscoring the distinct advantages associated with harnessing EVs as therapeutic agents independent of primitive stem cells [90].

Natural polysaccharides are biomolecules widely present in animals, plants, and microorganisms and exhibit remarkable biological activities [91]. Extensive basic research and clinical applications have demonstrated the safety and efficacy of polysaccharide-based hydrogel dressings for wound healing, making them an attractive option for wound management [92,93]. Polysaccharides possess various functional groups such as hydroxyl, carboxyl, and amine groups, which allow versatile chemical modifications to address their inherent limitations [94]. The combined application of natural polysaccharides or modified derivatives in conjunction with EVs not only enhances the mechanical strength and biocompatibility of hydrogel materials but also benefits the sustained release of active substances in EVs, ultimately producing a significantly enhanced pro-healing capacity.

### 3.2 Protein-based hydrogels

Proteins are vital biological macromolecules that consist of one or more polypeptide chains and serve as fundamental constituents of living organisms. Protein-based hydrogels, characterized by their multifunctional group structure and self-assembly properties, exhibit facile modification and crosslinking properties [95]. In contrast to hydrogels derived from chemically synthesized polymers, protein-based hydrogels offer distinct advantages including abundant availability, degradability, environmental friendliness, and broad applicability. Bioprotein-based hydrogels serve as versatile platforms that leverage the intrinsic attributes of natural proteins, such as their biocompatibility, biodegradability, non-toxicity, and cell adhesion properties. Furthermore, through the incorporation of polymers or functional substances, it is possible to further enhance the existing properties or introduce novel functionalities to these hydrogels [96].

Collagen, the predominant protein in the natural ECM, is the most abundant protein in mammals [97]. In the physiological milieu, collagen typically exists in a gel state and exhibits biomimetic hemostatic properties, effectively modulating cellular differentiation and migration. However, the utility of collagen is hindered by its inadequate mechanical properties and susceptibility to degradation by endogenous collagenases. Recently, recombinant collagen has emerged as a promising biomaterial prepared using synthetic biology techniques, with potential applications in diverse medical fields [98]. An illustrative example of the medical applications of recombinant collagen was demonstrated by Xu et al., who designed a novel rhCol III hydrogel loaded with EVs derived from hUC-MSCs. The release kinetics showed approximately 80% release of EVs within 10 days. *In vitro* evaluation showed that the rhCol III-EV composite hydrogel promotes the M2-type polarization of macrophages and the proliferation of fibroblasts and HUVEC cells, which is conducive to the maintenance of wound nutrients [73].

Gelatin, a denatured protein derived from collagen via mild hydrolysis, is a valuable derivative of collagen [99]. The molecular structure of gelatin comprises a multitude of reactive groups, enabling the preparation of hydrogels with diverse properties through modification, crosslinking, or compounding with other materials [100]. For instance, the introduction of methacryloyl groups to modify the amino side chains of gelatin results in the development of GelMA hydrogels, which exhibit photo crosslinking properties and significantly enhance the mechanical strength of the material. Consequently, GelMA hydrogels have emerged as promising materials for biomedical applications. In a notable study by Zhao et al., exosomes derived from HUVEC (HUVEC-exos) promoted skin regeneration by enhancing the proliferation and migration of keratinocytes and fibroblasts, which are critical effector cells for skin regeneration. Building on this finding, a wound dressing was designed by embedding HUVEC-exos within the GelMA hydrogel. The healing efficiency of the system was accessed in the full-thickness wound defects of rats. Faster wound closure and better angiogenesis were observed in animals treated with GelMA hydrogel loaded with HUVECs-exos [101].

Silk protein, a widely available, cost-effective, and sustainable natural material extracted from the silk of domestic silkworms, has been extensively used for the preparation of injectable hydrogels [94]. Shi et al. successfully fabricated a porous chitosan/silk protein hydrogel sponge via lyophilization (Fig. 1B). Scanning electron microscopy images confirmed the maintenance of an interconnected microporous structure in the hydrogel, featuring pore sizes ranging from 50 to 150 μm. These characteristics render this hydrogel suitable not only for wound dressings but also as a loading scaffold for exosomes. The effective loading of exosome particles was validated by observing the uniformly distributed green fluorescence from DiO-labeled GMSC-exos under laser confocal microscopy. *In vivo* studies demonstrated that the hydrogel-exosome group exhibited superior efficacy in promoting epithelial renewal, collagen deposition, and vascular regeneration compared with the control group using exosomes alone and the group employing hydrogel alone [35].

Although natural proteins exhibit exceptional attributes, single-protein hydrogels have certain limitations, including inadequate mechanical strength, specificity, and conformational flexibility [102]. To address these limitations and enhance the therapeutic efficacy, the incorporation of other polymers and the introduction of EVs have been explored to improve the physical properties and biological characteristics of dressings [103]. Despite the continuous optimization of protein-based hydrogels for the delivery of bioactive substances, most applications are still in the early stages of development and have yet to reach clinical implementation. Furthermore, the lack of mass-produced and cost-effective manufacturing technologies remains a significant hurdle that hampers the large-scale production and widespread application of protein-based hydrogels [104].

### 3.3 Synthetic hydrogels

#### 3.3.1 Polyethylene glycol

PEG, also known as polyethylene oxide, is a crystalline, thermoplastic, water-soluble polymer [105]. Hydrogels composed of PEG exhibit notable mechanical properties. PEG is considered biosafe and has received FDA approval for application in pharmaceutical formulations, food packaging, and other industries [106,107]. Kwak et al. developed an injectable PEG hydrogel (Exogel) loaded with M2 macrophage-derived exosomes (M2-exos) to facilitate the transition from M1 to M2 macrophage phenotypes (Fig. 1C). The balance of M1/M2-macrophages (Mφs) mediates the phase shift of inflammation-proliferation, which determines the quality of skin wound-healing outcomes. Whole-body fluorescence imaging in mice showed that exosomes encapsulated in hydrogels were more efficiently retained in the skin than free Exos, ensuring stable M2-Mφ polarization at the wound site. Immunohistochemistry and cytokine expression analyses indicated that the Exogel reservoir can provide a long-term supply of factors required to initiate and maintain the reprogramming of M1-Mφs into M2-Mφs, which contributes to wound healing [108].

#### 3.3.2 Polyvinyl alcohol

PVA is a biodegradable semi-crystalline synthetic polymer with moderate mechanical strength and favorable biocompatibility [109]. PVA hydrogels possess notable properties such as high permeability to small molecules, low interfacial tension, and high water content, making them suitable candidates for controlled drug release applications [110]. Nanohydrogels, characterized by dimensions smaller than 200 nm, have emerged as stable molecular delivery systems owing to their high drug-loading capacity, excellent permeability, and integration properties [111]. Zhang et al. encapsulated hUCMSC-exos in a nanogel composed of PVA and alginate (exo@H) to promote wound healing in diabetic rats (Fig. 1D). The therapeutic efficacies of nanohydrogel, exo, and exo@H were evaluated in a streptozotocin-induced diabetic rat model. The results demonstrated that both the exo and exo@H groups exhibited a significant reduction in the wound area compared to the nanohydrogel group. By postoperative day 18, the wounds in the exo@H group had almost completely closed, exhibiting intact epithelial tissue and a few hair follicles, whereas the wounds in the exo group remained visible. These findings suggested that the hydrogel effectively facilitates the targeted and efficient delivery of exosomes to the wound site, thereby enhancing its therapeutic potential for diabetic wound healing [50].

## 4 Mechanisms underlying the therapeutic effects of hydrogel-loaded EVs

Wound healing is a complex and dynamic process that involves a series of interconnected phases including hemostasis, inflammation, proliferation, and remodeling. These phases rely on the coordinated interactions between various cellular components, cytokines, and growth factors. In diabetic wounds, the healing process is often impaired owing to aberrant inflammation, elevated oxidative stress, impaired angiogenesis, delayed re-epithelialization, and increased susceptibility to infection. Consequently, there is growing interest in developing strategies to address these challenges and promote diabetic wound repair. One promising approach involves the use of EV-based hydrogel systems that provide a conducive microenvironment for wound healing while facilitating the sustained release of bioactive factors. Hydrogels, with their hydrophilic and cross-linked nature enabling controlled drug release, have been extensively explored as carriers for the topical delivery of EVs, which serve as vital paracrine products in wound healing. The pro-wound healing mechanism of EV-loaded composite hydrogels relies on the intrinsic properties of the hydrogels, bioactive substances contained within the EVs, and synergistic effects between the two. EV-loaded composite hydrogels can modulate various biomolecules, enabling them to achieve therapeutic goals, such as inflammation inhibition, antioxidant effects, antimicrobial activity, promotion of angiogenesis, and facilitation of tissue regeneration. The intricate interplay between the hydrogel matrix and the EVs within the composite system contributes to the overall efficacy of diabetic wound healing (Table 2).

**Table 2:** Pro-wound repair effect of hydrogel-loaded EVs from different material sources.

Polymers type	Source of encapsulant	Effects	Ref.
Chitosan-based hydrogels	BMSC- exos	anti-inflammatory and promotes angiogenesis	[78]
	hUCMSCs- exos	reduces inflammation	[112]
	hEnSC-exos	promotes angiogenesis and tissue granulation formation	[37]
Hyaluronic acid‒based hydrogels	M2 macrophages-exos	eradication of bacterial infection, alleviation of oxidative stress, stimulates angiogenesis and epithelization	[113]
	hMSC-exos	improves the proliferation, migration, and angiogenic ability	[79]
Alginate-based hydrogels	BMSC-EVs	inhibits excessive angiogenesis and collagen deposition	[62]
	ADSCs-exos	improves collagen synthesis and vessel formation	[85]
Collagen-based hydrogels	hUCMSCs-EVs	reduces the inflammatory response and promotes vascularization	[73]
	BMSCs-EVs	promotes collagen deposition	[90]
Gelatin-based hydrogels	platelet-EVs	inhibits inflammation and promotes angiogenesis	[114]
	hUCMSC-exos	promotes dermal fibroblast proliferation and migration	[115]
	periosteum-EVs	promotes angiogenesis	[116]
	BMSC-NVs	regulation of macrophage polarization	[117]
Silk fibroin-based hydrogels	PRP-exos	accelerates dermal angiogenesis	[108]
	hUCMSCs-exos	decreases inflammation	[118]
PEG-based hydrogels	ADSC-exos	promotes cell proliferation, enhances angiogenesis, and decreases the reactive oxygen species	[66,76,119]
	M2 macrophages-exos	promotes anti-inflammatory M2- macrophages polarization	[108]
PVA-based hydrogels	hUCMSCs-exos	promotes the proliferation, migration, and angiogenesis of the skin cell	[50]

### 4.1 Anti-inflammation

During the inflammatory phase of wound healing, Mφs play a critical role in both the clearance of apoptotic or necrotic cells from the injury site and the recruitment of vascular endothelial cells, fibroblasts, and keratinocytes through the release of various growth factors and cytokines [120]. Macrophages exhibit plasticity and display both proinflammatory (M1 phenotype) and anti-inflammatory (M2 phenotype) characteristics. In diabetic wounds, the persistence of chronic inflammation is partly attributable to an imbalance in macrophage M1/M2 polarization, wherein the transition from pro-to anti-inflammatory macrophages is unsuccessful [121]. Moreover, the hyperglycemic environment in diabetes stimulates macrophages to secrete proinflammatory cytokines, such as interleukin (IL)-1, IL-6, and tumor necrosis factor-α, further perpetuating the vicious cycle of M1 macrophage polarization and chronic inflammation [122,123].

**Fig. 2:**
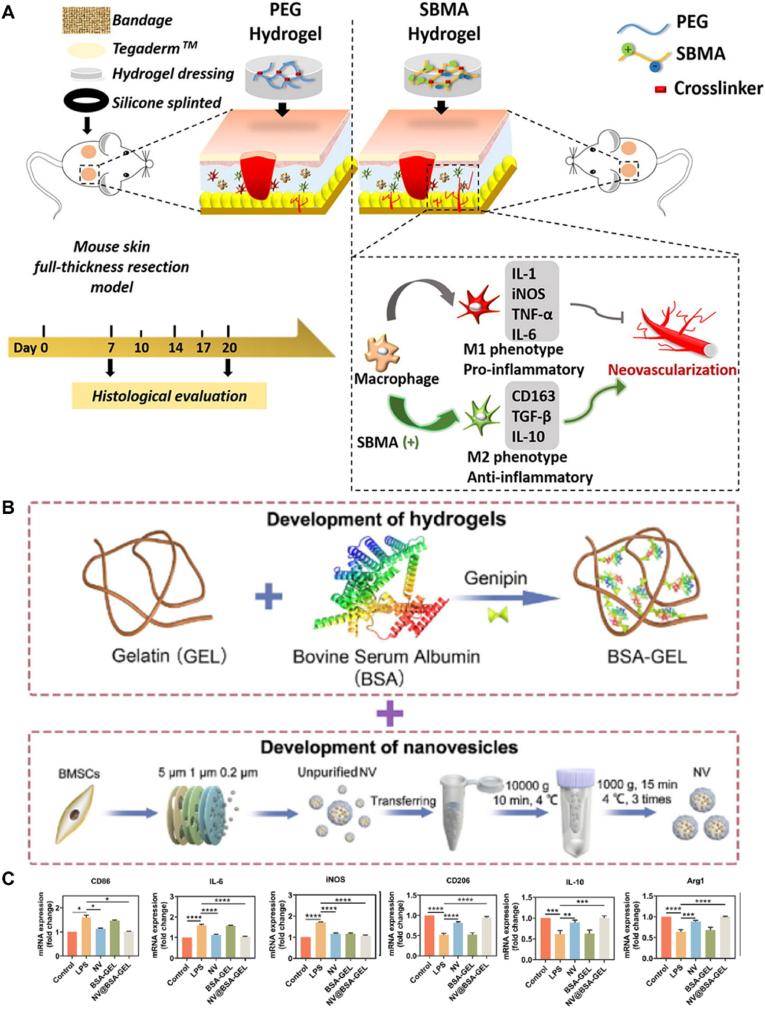
Anti-inflammatory mechanism of hydrogels loaded with EVs. (A) Schematic diagram of the poly (SBMA) hydrogel for wound closure. Reproduced with permission from Ref. [127], Copyright © 2018 Elsevier Ltd. (B) Schematic illustration of NV@BSA-GEL hydrogel fabrication. (C) Anti-inflammatory effects of the NV@BSA-GEL hydrogel on macrophages. Reproduced with permission from Ref. [117], Copyright © 2023 Elsevier Ltd. SBMA, sulfobetaine methacrylate.

Accumulating evidence suggests that hydrogels formulated with anti-inflammatory materials have the ability to protect and maintain a moist environment on the surface of wounds while simultaneously modulating the body's immune response and promoting the expression of anti-inflammatory factors [124] Currently, there are several hydrogel materials known for their anti-inflammatory properties, including natural polysaccharides, honey, and amphoteric materials [125,126]. For instance, zwitterionic sulfated poly (sulfobetaine methacrylate) hydrogels exhibit the dual functions of preventing the adhesion of harmful macromolecular substances and significantly reducing the inflammatory response by modulating inflammatory macrophages, thereby accelerating wound healing [127] (Fig. 2A). Additionally, hydrogels based on starPEG and glycosaminoglycan heparin derivatives effectively mitigate wound inflammation in patients with chronic ulcers by efficiently scavenging multiple inflammatory chemokines such as monocyte chemoattractant protein-1 and IL-8 [128].

To enhance the anti-inflammatory properties of hydrogels, significant attention has been directed toward the development of anti-inflammatory hydrogels incorporating biological components, as they offer the potential to directly interact with tissues and modulate inflammatory cells and factors. Among these, EVs have attracted considerable interest, particularly those derived from immune or stem cells [129,130]. Fan et al. used conductive hydrogels as a delivery system for bone marrow stem cell-derived exosomes (BMSC-exos). The exosomes exhibited high expression levels of miR199a, miR99a, miR146a, miR181a, and miR411, with miR199a showing the highest expression. Notably, miR199a acts as a regulator of the nuclear factor kappa-B pathway, thereby exerting a suppressive effect on inflammation [131]. Additionally, M2-exos exhibit remarkable cellular reprogramming abilities, facilitating the direct conversion of proinflammatory M1 macrophages to anti-inflammatory M2 macrophages at the injury site [132]. Zhang et al. demonstrated that hydrogel-loaded M2-exos further enhanced the expression level of arginase1 (Arg1), a protein associated with M2 macrophage polarization. Therefore, the hydrogel can serve as a reservoir for continuous stimulation of M2-Mφ polarization, thereby facilitating the resolution of acute inflammation [108].

Recently, Wu et al. prepared bioaugmented hydrogels composed of bovine serum albumin (BSA)-bridged gelatin loaded with BMSC-derived nanovesicles (NVs) using a one-pot method, named NV@BSA-GEL [117] (Fig. 2B and C). To evaluate the effect of the composite hydrogel on macrophages, Raw264.7 macrophages were stimulated with lipopolysaccharide (LPS) to induce the proinflammatory M1 phenotype. The M1 phenotypic marker CD86 was downregulated and the M2 phenotypic marker CD206 was upregulated in LPS-stimulated macrophages after treatment with NV and NV@BSA-GEL hydrogels, and the inflammatory cytokines IL-6 and nitric oxide synthase were significantly reduced in LPS-treated macrophages. In contrast, the expression of anti-inflammatory markers, such as Arg1 and IL-10, significantly increased after NV and NV@BSA-GEL hydrogel treatment. These results suggested that the NV and NV@BSA-GEL hydrogels can effectively inhibit the expression of proinflammatory cytokines in macrophages and promote macrophage polarization to the M2 phenotype. Additionally, Zhu et al. designed a composite hydrogel composed of GelMA and silk fibroin glycidyl methacrylate. This hydrogel served as a delivery platform for platelet-derived EVs and mesoporous silica nanoparticles loaded with resveratrol for the treatment of diabetic wounds [114]. Overall, both hydrogel types demonstrated enhanced anti-inflammatory effects by modulating the macrophage response and promoting the expression of anti-inflammatory factors, potentially offering a promising therapeutic approach for diabetic wounds.

In summary, anti-inflammatory hydrogels loaded with EVs show great potential for wound healing. These hydrogels not only benefit from the inherent anti-inflammatory properties of the hydrogel materials themselves but also leverage the immunomodulatory capabilities of the loaded EVs to effectively modulate the inflammatory response. It is important to simplify the preparation process of these hydrogels and enhance their safety to minimize potential side effects and optimize their anti-inflammatory effects. Streamlining manufacturing processes can facilitate large-scale production and clinical applications. By addressing these challenges and advancing the field, anti-inflammatory hydrogels loaded with EVs could be developed as effective therapeutic strategies for promoting wound healing and managing inflammatory conditions.

### 4.2 Antioxidation

**Fig. 3:**
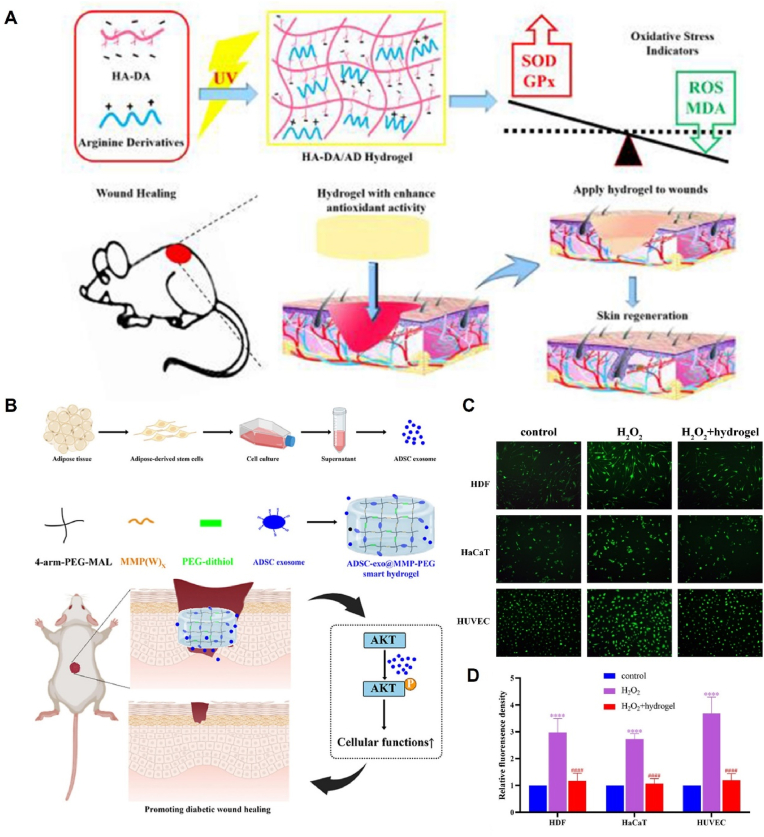
Antioxidant mechanism of hydrogels loaded with EVs. (A) HA-DA/hydrogel synthesis and antioxidant application. Reproduced with permission from Ref. [135], Copyright © 2019 Elsevier B.V. (B) Schematic illustration of the preparation and application of the ADSC-exo@MMP-PEG smart hydrogel. (C) Fluorescence images of ROS in HDFs, HaCaT cells, and HUVECs. (D) Statistical analysis of C. Reproduced with permission from Ref. [66], Copyright © 2022 Elsevier Ltd.

The excessive accumulation of reactive oxygen species (ROS) and oxidative stress can have detrimental effects on wound healing, leading to tissue damage and cell death. Hypoxia exacerbates the negative effects of oxidative stress on the healing process [133]. In this context, inhibition of oxidative stress has become a feasible and effective strategy for promoting wound healing. Antioxidative hydrogels are designed to scavenge ROS and mitigate the harmful effects of oxidative stress under pathological conditions. Hydrogels are formed by crosslinking one or more substances with ROS-scavenging properties [134]. One example is the dopamine-functionalized hyaluronic acid (HA-DA) hydrogel proposed by Zhang et al. Compared to conventional HA hydrogels, this hydrogel incorporates a novel antioxidant material, arginine derivative, which enhances its ROS-scavenging capabilities [135](Fig. 3A).

Hyperglycemia induces high intracellular oxidative stress, leading to cellular senescence and death. Previous investigations have shown that exosomes can repair oxidative stress-induced skin damage by adaptively regulating the nuclear factor-erythroid 2-related factor 2 (Nrf2) defense system. In skin injury, Nrf2 pathway activation produces a cascade effect, leading to the production of multiple strong antioxidants, including superoxide dismutase, glutathione peroxidase, and catalase [136]. Shiekh et al. constructed an oxygen-releasing antioxidant wound dressing (OxOBand) loaded with exosomes for diabetic wound healing. This dressing eliminates ROS through electron transfer and exhibits sustained oxygen release properties. As a result, OxOBand could further enhance the antioxidant response, leading to increased metabolic activity and cell viability compared to treatment with exosomes alone. *In vivo* experiments, immunostaining for 8-OHdG (a marker of oxidative stress) demonstrated a significant reduction in oxidative stress in wounds treated with OxOBand [137].

MMPs are a family of peptidases that degrade the ECM and are involved in tissue remodeling. Excessive ROS production can activate the overexpression of MMPs, which in turn hinders wound healing [138,139]. To address this issue, researchers have developed a smart hydrogel loaded with ADSC-exos that releases these exosomes in response to the diabetic wound microenvironment through the enzymatic degradation of MMP-2. *In vitro* experiments proved that high levels of intracellular ROS induced by hydrogen peroxide significantly affected the proliferation and migration of human dermal fibroblasts (HDFs), human keratinocytes (HaCats), and HUVECs. However, this weakened migration of cells was rescued by the smart hydrogel. These findings suggested that this smart hydrogel loaded with ADSC-exos can contribute to the healing of diabetic wounds by attenuating oxidative stress-induced damage to cells [66](Fig. 3B–D).

With in-depth research on the mechanisms of oxidative stress and wound healing, the application of antioxidant hydrogels has emerged as a prominent research focus in the field of wound dressing. Intelligent hydrogels capable of responding to the reactive oxygen microenvironment of wounds have been developed by incorporating antioxidant materials into hydrogel systems to facilitate wound healing. Future investigations should aim to explore the degradation kinetics of hydrogels used as carriers for EVs with controlled release, enabling the precise regulation of free radical scavenging and factor expression. This will ultimately facilitate the achievement of an intelligent balance of ROS, enhancing the therapeutic efficacy of antioxidant hydrogel-based approaches in wound healing applications.

### 4.3 Anti-infection

**Fig. 4:**
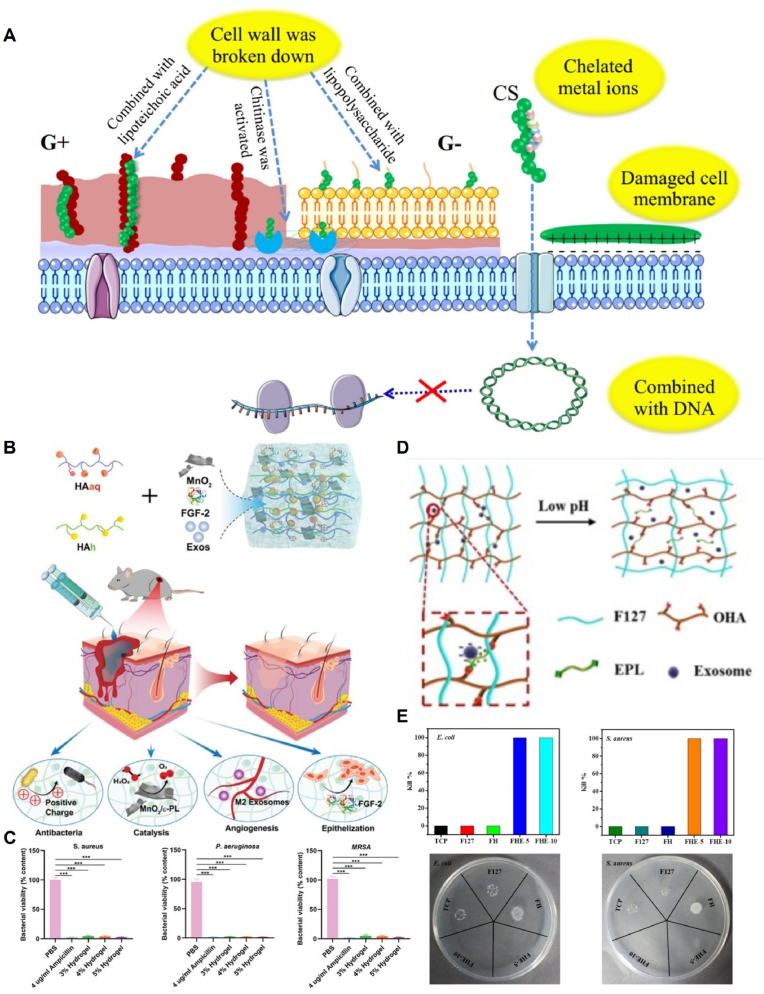
Anti-infection mechanism of hydrogels loaded with EVs. (A) Antibacterial mechanism of chitosan and its derivatives. Reproduced with permission from Ref. [143], Copyright © 2020 Elsevier B.V. (B) Construction and application of the multifunctional HA@ MnO2/FGF-2/Exos hydrogel. (C) Viability of *P. aeruginosa*, *S. aureus*, and MRSA after incubation for 4 h at 37 °C. Reproduced with permission from Ref. [113], Copyright © 2021 Wiley-VCH GmbH. (D) Scheme of pH-responsive exosome release in the FHE hydrogel. (E) Antimicrobial efficiency of FHE hydrogels. Reproduced with permission from Ref. [32], Copyright © Ivyspring International Publisher.

Infection is a significant factor impeding wound healing. Bacterial infections in wounds can lead to the accumulation of pus, resulting in delayed healing and potentially severe complications such as sepsis [140]. Consequently, the development of hydrogels with antimicrobial properties is crucial to combat infections. Hydrogels possess inherent antimicrobial properties that arise from their characteristics as skin barriers, which can absorb exudates and prevent bacterial entry, as well as their chemical composition containing antimicrobial components [14]. For example, chitosan-based dressings exhibit antimicrobial effects via two main mechanisms: disruption of bacterial cell membranes, inhibition of bacterial growth, and binding of positively charged chitosan to bacterial DNA, thereby hindering mRNA production and leading to bacterial death. However, simple chitosan hydrogels frequently face challenges in achieving satisfactory mechanical properties and potent antibacterial effects. The introduction of quaternary ammonium groups into the chitosan backbone is a commonly used modification method to overcome these limitations. This modification enhances the water solubility and imparts improved antimicrobial properties to the resulting hydrogels. The positively charged quaternary ammonium alkyl chains of the modified hydrogels were adsorbed onto the negatively charged bacterial surface and penetrated the bacterial cell wall. Moreover, the long hydrophobic alkyl chains of the quaternary ammonium compounds facilitate their adsorption onto the bacterial surface, ultimately leading to bacterial death [[141], [142], [143], [144]] (Fig. 4A).

The presence of a C=N double bond in the Schiff base moiety confers beneficial biological activities, including antibacterial, bactericidal, and antitumor properties, to Schiff base compounds. The acid-responsive nature of the Schiff base bond leads to its disruption in the presence of acids produced by bacterial metabolites. Consequently, this disruption causes the hydrogel to degrade, ultimately accelerating the release of antibiotics [145]. Xiong et al. evaluated the antibacterial capability of a hydrogel designated HA@MnO_2_, which consisted of manganese dioxide (MnO_2_) nanosheets and HA. The hydrogel was crosslinked via a Schiff base reaction between hydrazide-grafted HA (HAh) and aldehyde- and quaternary-ammonium-grafted HA (HAaq). The research findings revealed that the HA@MnO_2_ hydrogel displayed robust antibacterial activity against three commonly encountered bacteria associated with wound infections, namely, *Staphylococcus aureus* and methicillin-resistant *S. aureus* (MRSA), and gram-negative *Pseudomonas aeruginosa*, when compared to phosphate buffered saline. Moreover, the antimicrobial activity of HA@MnO2 hydrogel was comparable to that of ampicillin, which was mainly attributed to the positively charged quaternary ammonium groups on the HA backbone of HA@MnO2 hydrogel. Notably, an increase in the concentration of the HA@MnO_2_ hydrogel yielded a proportional augmentation in antibacterial activity, with the 5% hydrogel group exhibiting the most potent activity and a significant reduction in bacterial colonization. Furthermore, augmenting the HAh and HAaq content within the HA@MnO_2_ hydrogels significantly enhanced their antibacterial effects [113] (Fig. 4B and C).

Additionally, poly-ε-l-lysine (EPL) serves as a naturally occurring antimicrobial peptide derived from *Streptomyces albicans*, showcasing intrinsic antimicrobial activity. Wang et al. successfully engineered a multifunctional hydrogel incorporating ADSC-Exos, wherein the hydrogel scaffold, denoted as FHE, was composed of Pluronic F127 (PF127), oxidative hyaluronic acid, and EPL. Notably, EPL endowed the FHE hydrogel with potent antibacterial properties. Comparative evaluations with the F127 hydrogel demonstrated the remarkable effectiveness of the FHE hydrogel in eradicating both *Escherichia coli* and *S. aureus* after a 2-h incubation period. Conversely, incubation with F127-HA led to a substantial increase in the populations of *E. coli* and *S. aureus* [32] (Fig. 4D and E).

In summary, although there have been advancements in the development of antimicrobial hydrogels for the treatment of diabetic wounds, there remains a need for extensive research on the preparation of antimicrobial hydrogels and the selection of biomaterials. Further research is needed to incorporate different antimicrobial agents or materials into hydrogel matrices and explore innovative crosslinking methods, surface modifications, or load-responsive elements to create smart hydrogels that can selectively release antimicrobial agents in response to specific triggers such as pH or temperature changes. These investigations aimed to enhance the antimicrobial efficacy and biocompatibility of dressings, thus playing a crucial role in the treatment of trauma infections.

### 4.4 Pro-angiogenesis

Angiogenesis plays a crucial role in the wound healing process as it facilitates the delivery of oxygen, nutrients, and growth factors to injured tissues. The formation of microvascular networks involves multiple cell types and pro-angiogenic factors such as vascular endothelial growth factor (VEGF) and fibroblast growth factor (FGF) [146]. Recently, bionanotechnology has emerged as a promising approach for promoting angiogenesis in diabetic wounds. Bionic pro-angiogenic hydrogels can effectively modulate the stability, activity, and distribution of cytokines and growth factors. For instance, Lee et al. proposed the incorporation of microchannels within hydrogels to facilitate the perfusion of nutrients and oxygen, with the aim of optimizing the construction of bionic hydrogels. Hydrogels with microchannels can significantly promote the differentiation of BMSCs into endothelial cells, providing promising insights into angiogenesis [147].

EVs contain a variety of bioactive substances, such as proteins, DNA, RNA, and microRNAs, which have demonstrated promising effects in promoting angiogenesis in chronic wounds. For example, EVs derived from fibroblasts are enriched with pro-angiogenic miRNAs, such as miRNA-126, miRNA-130a, and miRNA-132, which stimulate the release of VEGF and FGF-2 in wounds. Similarly, exosomes derived from adipose-derived stem cells (ADSCs-Exos) promote angiogenesis by inhibiting the Notch1/Delta-like 4 pathway through miR-21. This inhibition leads to an increased expression of VEGF-A and hypoxia-inducible factor-1α (HIF-1α), both of which are critical for stimulating angiogenesis [148]. Furthermore, EVs from different sources activate various angiogenesis-related pathways such as the phosphatidylinositol 3-kinase (PI3K)/protein kinase B (Akt), HIF-1, and extracellular regulated protein kinases1/2 (ERK1/2) pathways, further contributing to the promotion of wound healing [[149], [150], [151]].

**Fig. 5:**
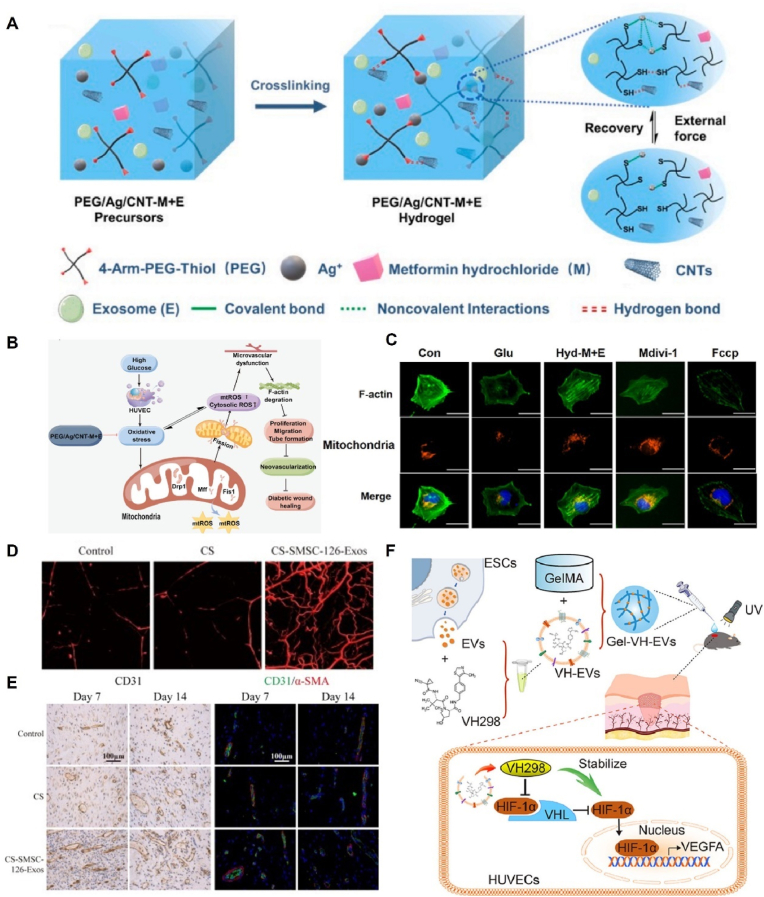
Pro-angiogenesis mechanism of hydrogels loaded with EVs. (A) Schematic of the preparation of the PEG/Ag/CNT-M + E hydrogel. (B) Schematic diagram showing the mechanism of microvascular injury protection by PEG/Ag/CNT-M + E hydrogels. (C) Coimmunofluorescence of mitochondria and F-actin. Reproduced with permission from Ref. [119], Copyright © 2023 Elsevier. (D) μCT images of angiogenesis treated with CS-SMSC-126-Exos. (E) Immunofluorescence staining of CD31 and α-SMA at 7 and 14 days after the operation. Reproduced with permission from Ref. [154], Copyright © 2016 Wiley Periodicals, Inc. (F) Formation of Gel-VH-EVs for pro-angiogenesis. Reproduced with permission from Ref. [155], Copyright © 2022 Elsevier Ltd.

Hydrogel-based delivery systems for EVs have demonstrated enhanced pro-angiogenic capacity by improving their retention and stability. Recently, Zhang et al. developed a novel strategy by designing dual-loaded self-healing hydrogels (PEG/Ag/CNT-M + E) incorporating exosomes and metformin to promote the healing of chronic diabetic wounds (Fig. 5A–C). This study demonstrated that the dual-loaded hydrogel formulation exerts beneficial effects by inhibiting mitochondrial fission, preserving F-actin homeostasis to protect microvascular function, and enhancing HUVEC migration and neointima formation [119]. Similarly, Mu et al. used peptide-modified viscous hydrogels loaded with hypoxia-stimulated exosomes (hypo-Exos). The increased HIF1-α content in exosomes under hypoxic conditions led to the upregulation of VEGF expression in targeted endothelial cells, thus promoting angiogenesis [152]. In addition to hypoxic stimulation, gene modification is another strategy for enhancing the pro-angiogenic properties of EVs. Exosomes derived from MSCs overexpressing miR-126–3p (SMSC-126-Exos) show significant pro-angiogenic effects; they promote the proliferation, migration, and tube formation of endothelial cells in a dose-dependent manner through activation of the PI3K/AKT and Mitogen-activated protein kinase pathways. Furthermore, the controlled release of SMSC-126-Exos using chitosan hydrogels resulted in a substantial increase in the number and density of neovascularized cells at the wound site. These findings highlight the potential of genetically modified EVs and hydrogel-based delivery systems for enhancing angiogenesis during wound healing [153,154] (Fig. 5D and E).

Stem cell-derived EVs have emerged as suitable vehicles for drug delivery. In a recent groundbreaking study, epidermal stem cell-derived EVs were used as a delivery platform for the small-molecule drug VH298 (VH-EVs). Notably, VH-EVs demonstrated remarkable pro-angiogenic effects both *in vitro* and *in vivo* by effectively stabilizing HIF-1α and activating the HIF-1α signaling pathway. This, in turn, accelerated the healing of diabetic wounds. To further enhance therapeutic efficacy, researchers have utilized photo crosslinked hydrogels with a controlled release of VH-EVs, which exhibit significant therapeutic effects in repairing skin defects. This EV-based drug delivery strategy holds great promise and introduces novel perspectives for the treatment of diabetic wounds [155] (Fig. 5F).

Pro-angiogenic hydrogels play a crucial role in enhancing wound healing by creating a favorable ECM environment for the growth of endothelial cells and modulating angiogenesis-related pathways through the controlled release of EVs. Nevertheless, further research is necessary to optimize the loading capacity of hydrogels and improve the controlled release of EVs, to attain improved synergistic effects and fully exploit their pro-angiogenic potential.

### 4.5 Cell proliferation

**Fig. 6:**
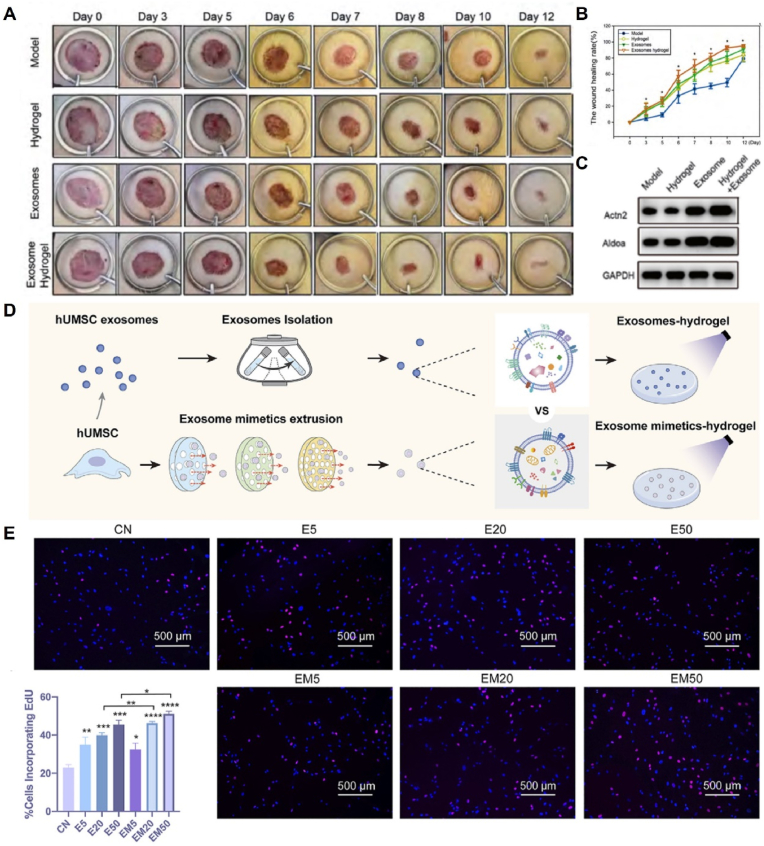
Pro-cell proliferation mechanism of hydrogels loaded with EVs. (A) Photographs of wound closure after intervention with the ADSC-*exo*-loaded β-ChNF hydrogel. (B) Wound closure rates of the indicated treatment groups. (C) Expression of Actn2 and Aldoa protein in rat skin. Reproduced with permission from Ref. [156], Copyright © 2022 VIA Medicine Journals. (D) Schematic diagram of the hUMSC-EM hydrogel for skin wound healing. (E) hUMSC-derived EMs promoted the proliferation of hDF-a. Reproduced with permission from Ref. [115], Copyright © 2022 Frontiers.

During the proliferative phase, a large number of keratinocytes and fibroblasts proliferate and migrate owing to stimulation by growth factors, thus promoting wound re-epithelialization. EVs contain various active molecules such as mRNAs, miRNAs, and growth factors, which can influence other cells and regulate multiple biological processes. For example, ADSC-derived EVs affect keratinocyte migration and accelerate reepithelialization by activating the AKT/glycogen synthase kinase 3 β pathway. Furthermore, ADSC and BMSC-EVs show dose-dependent stimulation of the cell cycle, growth, and migration of human dermal fibroblasts in chronic diabetic wounds. However, the regenerative healing capacity of EVs is limited owing to their short lifespan. To address this limitation, hydrogel-EV complexes can be used to delay the release of EVs and create a temporary ECM that facilitates cell infiltration and adhesion. In a study by Liu et al., β-chitin nanofiber (β-ChNF) hydrogels were utilized as effective carriers of ADSC-exos to promote wound healing. Based on transcriptomic and proteomic analyses, 18 differentially expressed genes and 169 proteins were identified. Further mechanistic studies revealed that in the ADSC-*exo*-loaded β-ChNF hydrogel treatment group, the complement factor D (CFD) protein was significantly downregulated, whereas its downstream alpha-actinin-2 (Actn2) and aldolase A (Aldoa) protein expressions were increased. Increased Aldoa expression promotes cell migration by enhancing the expression of intercellular adhesion-related proteins. These findings confirmed that β-ChNF hydrogels loaded with ADSC-exos have the potential to promote fibroblast proliferation by regulating the expression of CFD and downstream Aldoa proteins (Fig. 6A–C) [156].

Exosome mimics (EMs) have recently gained significant attention in the field of tissue regeneration as engineered nanoparticles that offer advantages in terms of availability and cost-effectiveness [157]. In a study conducted by Zhu et al., the effects of GelMA-loaded exosomes and exosome mimics were compared for their potential in wound healing (Fig. 6D and E). These results demonstrated that the exosome mimics promoted the proliferation and migration of dermal fibroblast (hDF-a) cells *in vitro*. Interestingly, the GelMA-EM group exhibited better wound healing efficacy than the GelMA-Exo group. To gain insight into the mechanism of action of exosome mimics, researchers performed proteomic analyses, which revealed that exosome mimics may enhance wound healing by boosting the expression of mitochondria-derived oxidative phosphorylation-related proteins in wound cells [115]. In another study, a wound dressing was developed using a composite hydrogel composed of silk fibroin (SF) nanofibers and alginate-loaded exosomes (SF-ALG-Exos). The SF-ALG-Exos hydrogel exhibited a suitable water vapor transfer rate and effectively prevented infection by minimizing leakage between the dressing and skin. *In vitro* studies showed that SF-ALG-Exos promotes cell-matrix interactions and stimulates tissue repair by enhancing fibroblast proliferation [158].

EV-loaded composite hydrogels hold significant promise in promoting diabetic wound healing and offer strong evidence for future clinical applications. By leveraging the principles of wound healing, various functional hydrogels can be designed and chosen to serve as mechanical barriers for the wound while delivering the necessary active substances at different stages of the healing process. This approach offers excellent prospects for the application of hydrogels to diabetic wound healing. The ability to precisely control the release of therapeutic agents and create an environment conducive for tissue regeneration makes hydrogels valuable tools for promoting wound healing and improving outcomes in diabetes patients.

## 5 Conclusions and future perspectives

Diabetic wound healing remains a serious challenge due to its complex pathophysiologic mechanisms. Recent advancements in biomaterials have introduced a novel approach that utilizes composite materials with EV-loaded hydrogels to treat diabetic wounds. This review aimed to elucidate the potential application of hydrogel-EV composites by highlighting their binding strategies, the effects of hydrogels from different material sources on diabetic wounds, and their healing mechanisms. Due to the limitations of hydrogels alone in meeting the complex needs of wound repair and the rapid clearance of directly injected EVs from the body, the strategy of encapsulating EVs within hydrogels capitalizes on their strengths while mitigating their weaknesses, showing great potential for accelerating the healing of diabetic wounds.

With advancements in synthesis and preparation techniques, a range of multifunctional hydrogel dressings capable of regulating the microenvironment of chronic diabetic wounds has been developed. However, challenges remain in achieving the stable and scalable production of hydrogel dressings for clinical applications. From a materials standpoint, hydrogels based on natural biomaterials often exhibit inadequate mechanical properties, necessitating chemical modification or 3D printing to enhance their physicochemical properties. Nevertheless, the presence of residual monomers or crosslinking agents during the preparation process poses challenges to material stability and biosafety, as they become trapped within the hydrogel matrix. From a functionality standpoint, incorporating bioactive materials such as EVs or nanomaterials into hydrogels can confer improved biological properties and enable the controlled release of EVs to enhance therapeutic effects. However, research on the loading efficiency and release kinetics of EVs, particularly *in vivo*, is limited. Furthermore, it is worth noting that the gelation process of temperature-sensitive hydrogels and the optimal storage temperatures for extracellular vesicles (EVs) vary significantly, which presents an additional challenge in their clinical translation [159]. Consequently, future studies should explore suitable hydrogel types for EV delivery and innovatively develop hydrogels with customizable physicochemical properties as platforms for achieving controlled *in vivo* release rates of EVs.

Currently, the main sources of EVs loaded in hydrogels include adipose stem cells, mesenchymal stem cells, macrophages, and platelets. Among them, MSC-derived EVs are the most studied. By encapsulating EVs within hydrogels, it is possible to improve the microenvironment of chronic wounds at various levels, including reducing the expression or activity of inflammatory factors, decreasing ROS levels, eliminating infection, and promoting vascularization and tissue regeneration. Moreover, it is worth noting that extracellular vesicle-based therapies hold substantial promise in diverse domains, including but not limited to cancer immunotherapy [160], cardiovascular disease [161], and renal disorders [162]. However, EVs also have inherent limitations, such as a short circulating half-life and low concentration of functional molecules. Additionally, mass production of EVs remains a significant obstacle hindering their clinical application. In order to surmount these constraints and streamline the clinical applicability of EV-based therapies, forthcoming research endeavors ought to concentrate on the formulation of standardized protocols for the isolation, purification, and storage of EVs. Simultaneously, there is a need to delve deeper into the elucidation of the fundamental mechanisms underpinning the action of EVs in the context of chronic wound healing. Furthermore, future investigations should endeavor to explore the synergistic potential of combining diverse biomaterials with EVs to address the intricacies of disease microenvironments more comprehensively.

Although hydrogels have great potential as advanced wound dressings, the limited availability of clinically approved products highlights the need for additional clinical trials and *in vivo* studies. Diabetic wounds exhibit intricate and diverse characteristics, underscoring the critical necessity for personalized hydrogel dressing designs. Tailoring these dressings to individual patient variations and trauma-specific conditions is imperative to attain the highest level of therapeutic effectiveness. Furthermore, the use of EVs and the integration of electronic devices can enhance the therapeutic effectiveness and monitoring capabilities of hydrogel dressings. Future research should prioritize the development of multifunctional and responsive wound dressings, thereby extending the horizons of hydrogel innovation beyond chemical and biological functionalities. Such advancements in hydrogel technology hold the potential to significantly enhance wound healing outcomes and enable the delivery of personalized treatments.

## Declaration of competing interest

The authors declare that they have no known competing financial interests or personal relationships that could have appeared to influence the work reported in this paper.

## Data Availability

Data will be made available on request.
